# Evaluating accuracy and reproducibility of ChatGPT responses to patient-based questions in Ophthalmology: An observational study

**DOI:** 10.1097/MD.0000000000039120

**Published:** 2024-08-09

**Authors:** Asem A. Alqudah, Abdelwahab J. Aleshawi, Mohammed Baker, Zaina Alnajjar, Ibrahim Ayasrah, Yaqoot Ta’ani, Mohammad Al Salkhadi, Shaima’a Aljawarneh

**Affiliations:** a Faculty of Medicine, Jordan University of Science and Technology (JUST), Irbid, Jordan; b Faculty of Medicine, Hashemite University, Zarqa, Jordan.

**Keywords:** artificial intelligence, ChatGPT, glaucoma, ophthalmology

## Abstract

Chat Generative Pre-Trained Transformer (ChatGPT) is an online large language model that appears to be a popular source of health information, as it can provide patients with answers in the form of human-like text, although the accuracy and safety of its responses are not evident. This study aims to evaluate the accuracy and reproducibility of ChatGPT responses to patients-based questions in ophthalmology. We collected 150 questions from the “Ask an ophthalmologist” page of the American Academy of Ophthalmology, which were reviewed and refined by two ophthalmologists for their eligibility. Each question was inputted into ChatGPT twice using the “new chat” option. The grading scale included the following: (1) comprehensive, (2) correct but inadequate, (3) some correct and some incorrect, and (4) completely incorrect. Totally, 117 questions were inputted into ChatGPT, which provided “comprehensive” responses to 70/117 (59.8%) of questions. Concerning reproducibility, it was defined as no difference in grading categories (1 and 2 vs 3 and 4) between the 2 responses for each question. ChatGPT provided reproducible responses to 91.5% of questions. This study shows moderate accuracy and reproducibility of ChatGPT responses to patients’ questions in ophthalmology. ChatGPT may be—after more modifications—a supplementary health information source, which should be used as an adjunct, but not a substitute, to medical advice. The reliability of ChatGPT should undergo more investigations.

## 1. Introduction

Artificial intelligence (AI) is a simulation of human intelligence that thinks and learns like a human.^[1]^ AI has grown significantly in recent years and become integrated into numerous products and services for which individuals have been increasingly relying on it.

In November 2022, Chat Generative Pre-Trained Transformer (ChatGPT) was developed by OpenAI.^[2]^ ChatGPT is an AI-based language model developed to generate human-like text responses mimicking a conversation, making it suitable for various aspects of life.^[3]^ The particular reason why many individuals rely on ChatGPT is because it is accessible, easy to use, and informative, especially for medical concerns.

Although ChatGPT has brought significant benefits to people’s lives, the increased reliance on it brings about several consequences. Specifically, as patients turn to these AI applications for guidance on their healthcare needs, the accuracy and reliability of the information provided become critical.^[4]^ Recent studies examined ChatGPT applications in healthcare settings. Yeo et. al. examined ChatGPT’s responses in cirrhosis and hepatocellular carcinoma which revealed response accuracy rates of 79.1% and 74.0% for cirrhosis and hepatocellular carcinoma respectively, suggesting its role more as a supplementary tool than a primary healthcare resource.^[4]^ A similar role was demonstrated with patients with diabetes using ChatGPT as an educational tool for diabetes-related queries.^[5]^ For example, chronic conditions that are generally stable and require ongoing management might be well-suited to the knowledge ChatGPT is trained on. In contrast, diseases with more nuanced or unpredictable outcomes may present greater challenges for accurate AI-guided advice.^[6]^

Many studies have evaluated ChatGPT responses in ophthalmology. Antaki et. al. used two 260-question simulated exams from the Basic and Clinical Science Course (BCSC) Self-Assessment Program and the OphthoQuestions online question bank to compare the accuracy of responses generated by different versions of ChatGPT (3.5).^[7]^ They found that the legacy model achieved 55.8% accuracy on the BCSC set and 42.7% on the OphthoQuestions set. With ChatGPT Plus, accuracy increased to 59.4% and 49.2%, respectively.^[7]^ On the other hand, Bernstein et al used patients’ questions that were answered by the American Academy of Ophthalmology (AAO)-affiliated ophthalmologists to evaluate the quality of ophthalmology advice generated by an LLM chatbot in comparison with ophthalmologist-written advice, with the assistance of a masked panel of 8 board-certified ophthalmologists.^[8]^ The average ratio of accuracy for distinguishing between AI and human responses was 61.3%. Of 800 evaluations of chatbot-written answers, only 168 answers (21.0%) were marked as human-written, while 517 of 800 human-written answers (64.6%) were marked as AI-written, which shows the high ability of ChatGPT (version 3.5) to simulate the human texts written by ophthalmologists.^[8]^

However, data on ChatGPT accuracy and reproducibility in patient-based questions are lacking. Therefore, this study aims to measure the accuracy of ChatGPT responses to patients’ concerns about ophthalmology conditions. Given the specialized nature of ophthalmology, which often involves a complex interplay of symptoms, treatments, and patient lifestyle factors, this study seeks to understand how well ChatGPT can handle such specific medical inquiries using questions obtained from “Ask an ophthalmologist” page of the AAO.

## 2. Methods

Institutional review board approval was not required for this type of articles.

### 2.1. Question curation/data source

Questions were first obtained from the “Ask an ophthalmologist” page of the AAO Then, 2 authors selected, reviewed, and approved the questions to evaluate their inclusion in the study. Questions were then further evaluated, to exclude duplicates and irrelevant questions. Questions of a general nature that necessitated subjective or personalized responses were likewise excluded. Some questions were grammatically edited to ensure comprehensibility. To conduct statistical analysis, questions were categorized into 7 ophthalmology-related categories to assess ChatGPT’s performance efficiently: (1) glaucoma; (2) cataract; (3) infectious disorders; (4) astigmatism; (5) retinal disorders; (6) LASIK and laser procedures; (7) strabismus and amblyopia. Finally, 115 questions were used to generate responses from ChatGPT.

### 2.2. Response generation

Each question was prompted to ChatGPT (3.5 version) twice. Each entry was on separate occasions using the “new chat” function with the goal of generating 2 responses per question. This was done to determine the reproducibility of responses to the same question.

### 2.3. Question grading

Responses to questions were first independently graded for accuracy and reproducibility by 2 board-certified ophthalmologist reviewers. Reviewers were instructed to grade the accuracy of responses based on known information leading up to 2021. Reproducibility was graded based on the similarity in accuracy of the 2 responses per question generated by ChatGPT. If the responses were similar, direct measurement of the similar grade was obtained. If the responses were not similar, the first response was utilized for grading. In this way, we obtain both accuracy and reproducibility.

Accuracy of each response was graded with the following scale:

1. Comprehensive: Defined as accurate and comprehensive, nothing more a board-certified ophthalmologist can add if asked this question by a patient.
2. Correct but inadequate: All information is correct but incomplete; a board-certified ophthalmologist would have more important information to add if asked this question by a patient.
3. Some correct and some incorrect.
4. Completely incorrect.

Disagreement in reproducibility or grading of each response was resolved by a meeting to reach consensus between the 2 board-certified ophthalmologist reviewers. The final grades were then compiled and used to analyze the overall performance of ChatGPT in answering questions related to ophthalmology.

### 2.4. Statistical analysis

Extracted data were entered into a spreadsheet. Statistical analysis was performed using the IBM SPSS statistical package for Windows v.26 (Armonk, NY). Data was expressed as frequency (percentage) for nominal data. Proportions of responses earning each grade were calculated. To determine reproducibility, responses were categorized into 2 groups: a grade of 1 and 2 comprised the first group, and a grade of 3 and 4 comprised the second group. The 2 responses to each question were considered significantly different from one another, or not reproducible if the assigned grades for each response fell under different groups.

## 3. Results

Totally, 115 questions were inputted into ChatGPT (see File S1, Supplemental Digital Content, http://links.lww.com/MD/N283). ChatGPT provided “comprehensive” responses to 70/117 (59.8%) of questions. In relation to categories, the model provided “comprehensive” responses to 64.7% of questions related to “Glaucoma,” 60% of questions related to “Cataract,” 73.3% of questions related to “infectious disorders and conjunctivitis,” 57.9% to questions about “Astigmatism,” 60% to questions about “LASIK and Laser procedures,” and 70% to questions related to “Amblyopia and strabismus” (Table 1). On the other hand, the percentage of comprehensive responses provided to questions related to retinal diseases was the lowest, with only 50% of questions being answered comprehensively, and 30.5% of questions being answered by responses graded as “Correct but incomplete.” Overall, only one question under the “retinal diseases” category was provided with a “completely incorrect” response, which is “Does drinking water eliminate flashes?.”

**Table 1:** Grading of responses generated by ChatGPT to questions related to ophthalmology categorized by question type.

	Comprehensive	Correct but incomplete	Some correct and some incorrect	Completely incorrect
Glaucoma (N = 17)	64.7%	35.3%	0.0%	0.0%
Cataract (N = 10)	60%	20%	20%	0.0%
Infectious disorders (N = 15)	73.3%	20%	6.7%	0.0%
Astigmatism (N = 19)	57.9%	31.6%	10.5%	0.0%
Retinal disorders (N = 36)	50%	30.5%	16.6%	2.7%
LASIK and laser procedures (N = 10)	60%	40%	20%	0.0%
Strabismus and amblyopia (N = 10)	70%	28.2%	11.1%	0.0%
Overall accuracy (%)	59.8%	28.2%	11.1%	0.8%

ChatGPT = Chat Generative Pre-Trained Transformer, N = number, SD = standard deviation.

In relation to reproducibility, it was defined as no difference in grading categories (1 and 2 vs 3 and 4) between the 2 responses for each question. ChatGPT provided reproducible responses to 91.5% of questions. Responses were reproducible to 100% of questions under the categories “Cataract” and “LASIK & Laser procedures,” while it was lower in other categories (Table 2).

**Table 2:** Proportion of questions with reproducible responses generated by ChatGPT categorized by question type.

Category	Percentage of reproducibility
Glaucoma (N = 17)	94.1%
Cataract (N = 10)	100%
Infectious disorders (N = 15)	93.3%
Astigmatism (N = 19)	94.7%
Retinal disorders (N = 36)	86.1%
LASIK and laser procedures (N = 10)	100%
Strabismus and amblyopia (N = 10)	80%

ChatGPT = Chat Generative Pre-Trained Transformer, N = number.

## 4. Discussion

In this study, we evaluated the accuracy and reproducibility of ChatGPT responses to patients’ concerns using patients-written questions from the AAO. We found that ChatGPT responded comprehensively to 59.8% of the questions, with a reproducibility rate of 91.5 %. These findings suggest a high accuracy and reproducibility to patients’ questions in ophthalmology.

ChatGPT is an AI-based chatbot developed to generate human-like text responses and is trained on a large database of information from a wide range of sources including online websites, books, and articles leading up to 2021. The model was brought into the limelight because it made the process of interacting with AI simple, accessible, and free. It can be used to answer questions, hold conversations, improve, or review academic writing, and develop study plans.^[5]^ It is found to have a potential use of assisting decision-makers in healthcare in summarizing relevant guidelines and treatment options with potential benefits, side effects, and drug interactions.^[5]^ ChatGPT has its own limitations, for example, given one phrasing of a question, the model can claim to not know the answer, but given a slight rephrase, can answer correctly.^[6]^ More studies need to be conducted to fully understand how to navigate ChatGPT’s strengths and limitations in different medical specialties.

Our findings align with the existing literature about ChatGPT performance in ophthalmology. Antaki et.al tested 2 versions of ChatGPT (based on version 3.5) on 2 question banks related to board examination in ophthalmology, and showed that legacy model achieved an accuracy of 55.8% on the BCSC set and 42.7% on the OphthoQuestions set, whereas ChatGPT Plus achieved 59.4% correct response rate on the BCSC set and 49.2% on the OphthoQuestions.^[7]^ In addition, Taloni et al found that ChatGPT 4.0 answered correctly 82.4% of the questions in the self-assessment program of American Academy of Ophthalmology, which was higher than human scores (75.7%).^[9]^ It was also shown that ChatGPT performs accurately when responding to questions about orbital and oculofacial disorders, with an average appropriateness score of 5.3/6.0 (“mostly appropriate” to “completely appropriate”).^[10]^

Our study found that ChatGPT scored best in the infectious disorders section (73.3%), and poorest in the retinal disorders section (50%). Antaki et al showed that the legacy model performed best in general medicine (75%), fundamentals (60%), and cornea (60%), but as well in glaucoma, (37.5%), and pediatrics and strabismus (42.5%), and neuro-ophthalmology (25%),^[7]^ which is in contradiction to Madadi et al findings that showed the potential to diagnose cases related to neuro-ophthalmology with comparable accuracy to certified neuro-ophthalmologist, with estimated accuracy of 59% and 82% for ChatGPT 3.5 and ChatGPT 4.0, respectively.^[11]^ Furthermore, although ChatGPT Plus model showed the same strength in same subjects as the legacy model, it’s poorest section remained neuro-ophthalmology, in addition to oculo-plastics and clinical optics.^[7]^

We used patients-written questions to simulate the usage of ChatGPT 3.5 by patients when they seek information regarding their medical condition, as it’s availability will possibly make ChatGPT a popular source pf information among patients. Similarly, Bernstein et al (version 3.5) used 200 question from The Eye Care Forum of the AAO to evaluate the ability of ChatGPT to simulate the answers written by AAO-affiliated ophthalmologists.^[8]^ Their 8 members panel accurately distinguished between AI and human responses with average accuracy of 61.3%, where 21% of the 800 evaluations of chatbot-written answers were marked as human-written.^[8]^ In addition, they found that the possibility of ChatGPT to include incorrect or inappropriate content in its answers were comparable with human answers, which applies in similar way to the likelihood of harm and its extent.^[8]^ ChatGPT was also evaluated using patients-based questions in other medical domains. In bariatric surgery, Samaan et al found that 86.8% of ChatGPT responses to questions were “accurate and comprehensive, with a reproducibility rate of 90.7%.^[12]^ On the other hand, Yeo et al (using 3.5 version) found that only 79.1% of responses were correct and 47.3% were comprehensive on questions related to cirrhosis, compared to 74.0% correct and 41.1% comprehensive responses on questions on hepatocellular carcinoma.^[4]^ These findings suggest that ChatGPT can be a very effective source of information and an adjunct to the medical advice, but not a substitute. Given the fact that many patients seek health information from an online sources, it’s very likely that high proportions are using LLM chatbot models as a source for medical advice about their medical conditions.^[13]^

Many studies suggest that high levels of health literacy among patients is associated with better care and outcomes regarding their illnesses. It can do so by increasing disease awareness and compliance with their treatments, higher surveillance for some complications, and decreased medical expenses.^[14]^ Same goes for literacy regarding eye-related diseases.^[15,16]^

There are several ways patients can seek information about their illnesses. One common way is by using the search engines on the internet.^[17]^ They are informative to some extent, however, search results can be overwhelming and misleading, as they provide dozens of websites that are related to the question, but without a direct comprehensive answer. The other brand-new way of providing information is ChatGPT. It is a free tool that provides a potentially reliable and accurate health information in a smooth conversation with the patient.^[2]^

Since ChatGPT was launched in November 2022, its use by patients seeking health information is on the rise, since it has numerous advantages over the classic search engines. For instance, it provides you with the details of your illness in a comprehensive conversational dialogue, which can contribute to reduced health literacy, reduce unnecessary anxiety among patients regarding their illnesses. In addition, it is free-of-charge, which makes it accessible for patients with financial limitations, which are already more prone to poor health outcomes.^[18]^ Furthermore, it can help improve patient outcomes by providing personalized care plans based on individual needs. It can even show empathy in its responses to patients and their caregivers and offer feasible recommendations for better outcomes.

On the other hand, ChatGPT has some drawbacks that make it less reliable. One is being trained on information only up until 2021, thereby providing some information that is outdated. The second is that the dataset it uses to produce answers in unknown, which may affect its reliability. However, ChatGPT is improving with time, and its drawbacks can be resolved later. As for our study, ChatGPT is a reliable source of information for ophthalmology patients. However, till the time of this article, it should not replace the seeking for professional medical advice.

AI in healthcare is a promising technology with enhanced diagnostics, streamlined processes, and improved patient care. However, this technology is accompanied by certain ethical implications that demand important consideration. Privacy and data security are paramount concerns, necessitating robust anonymization techniques to protect patient data. Algorithmic bias poses a significant challenge, demanding diverse datasets and ongoing monitoring to ensure fairness. Transparency and explainability in AI decision-making processes enhance trust and accountability. Ethics must remain at the forefront in the ever-evolving realm of healthcare technology. By embracing these strategies and best practices, healthcare systems and professionals can harness the potential of AI, ensuring responsible and ethical integration that benefits patients while upholding the highest ethical standards.^[19]^

### 4.1. Strengths and limitations

To the best of our knowledge, this is one of the few studies to examine the utility of the model ChatGPT in the field of ophthalmology. Patient questions were obtained from a famous reliable source—the “Ask an ophthalmologist” page of the AAO to provide a comprehensive and realistic sample of patient questions. Responses to questions were first independently graded for accuracy and reproducibility by 2 board-certified ophthalmologist reviewers to comprehensively evaluate the accuracy and reproducibility of ChatGPT’s responses. However; our study has some limitations. Firstly, we have relatively used a smaller number of questions compared to previous studies, which may affect the way the results can show the effectivity of ChatGPT. Secondly, the source of information in which ChatGPT was trained on is unknown which may impact the reliability of its responses for certain topics. Third, medical guidelines and standards of practice differ from country to country according to the relevant medical society, which makes it difficult to generalize these results to each user in every country. lastly, we used one version of ChatGPT only (GPT 3.5), which has lower abilities than advanced versions, such as ChatGPT 4.0. We run the study on this version because it is the free version that is accessible for public.

## 5. Conclusion

The large language model ChatGPT provided relatively moderate accuracy and reproducibility responses to common questions related to ophthalmology. ChatGPT still is not reliable to obtain major healthcare information; rather; it is helpful as a supplementary healthcare information source. ChatGPT may serve as a helpful adjunct, but not an exclusive, source of information regarding eye-related diseases. We encourage future studies to examine how to utilize this technology to improve patient outcomes and quality of life. The main healthcare resources should not be replaced.

## Author contributions

**Conceptualization:** Asem A. Alqudah, Abdelwahab J. Aleshawi, Ibrahim Ayasrah, Yaqoot Ta'ani, Mohammad Al Salkhadi, Shaima'a Aljawarneh.

**Data curation:** Asem A. Alqudah, Mohammed Baker, Ibrahim Ayasrah, Shaima'a Aljawarneh.

**Formal analysis:** Mohammed Baker, Mohammad Al Salkhadi.

**Investigation:** Asem A. Alqudah, Mohammed Baker, Zaina Alnajjar, Ibrahim Ayasrah, Mohammad Al Salkhadi.

**Methodology:** Asem A. Alqudah, Mohammed Baker, Mohammad Al Salkhadi, Shaima'a Aljawarneh.

**Resources:** Asem A. Alqudah, Mohammed Baker.

**Software:** Asem A. Alqudah, Mohammed Baker, Ibrahim Ayasrah, Yaqoot Ta'ani.

**Supervision:** Asem A. Alqudah, Zaina Alnajjar.

**Validation:** Asem A. Alqudah, Abdelwahab J. Aleshawi, Mohammed Baker, Zaina Alnajjar, Ibrahim Ayasrah, Yaqoot Ta'ani, Mohammad Al Salkhadi, Shaima'a Aljawarneh.

**Visualization:** Asem A. Alqudah, Abdelwahab J. Aleshawi, Mohammed Baker.

**Writing – original draft:** Asem A. Alqudah, Abdelwahab J. Aleshawi, Zaina Alnajjar, Yaqoot Ta'ani, Shaima'a Aljawarneh.

**Writing – review & editing:** Abdelwahab J. Aleshawi, Zaina Alnajjar, Ibrahim Ayasrah, Mohammad Al Salkhadi, Shaima'a Aljawarneh.

## Supplementary Material
