# Supplementary material for: Evaluating accuracy and reproducibility of ChatGPT responses to patient-based questions in Ophthalmology: An observational study
Source: Medicine (Baltimore). 2024 Aug 9;103(32):e39120. doi: 10.1097/MD.0000000000039120 (PMC11315477; doi:10.1097/MD.0000000000039120)
Supplement: Supplementary file 1 [file medi-103-e39120-s001.docx]

**Ophthalmology FAQs (115)**

**Glaucoma / 17**

1. **How does using antihistamines affect my glaucoma?**

- **Answer:**
- Antihistamines can cause an angle closure glaucoma attack (when fluid is completely blocked from leaving the eye, causing a dangerous rise in pressure) in some patients who have narrow angles. You should check with your ophthalmologist to learn what type of glaucoma you have and whether antihistamine usage could cause this problem.

1. **Is ibuprofen safe to use for pain relief if I’m a glaucoma patient?**

- **Answer:**
- There is no evidence that ibuprofen or other NSAIDs (non-steroidal anti-inflammatory drugs) have any impact on the internal pressure of the eye which is the issue with glaucoma. Therefore, it is safe to use this class of drugs even if you have glaucoma. However, please discuss use of ibuprofen and all other medications with your doctor.

1. **Is it safe to take prescription stimulants with glaucoma?**

- **Answer:**
- If you have open angle glaucoma, then stimulants such as Adderall are considered safe. But if you have angle closure (or narrow angle) glaucoma, this type of medication can increase the risk of a sudden increase in eye pressure (acute angle closure) and possible vision loss. The risk of this happening varies from person to person, so you need to discuss this with your ophthalmologist.
- An in-person exam will help determine whether taking medications like Adderall is safe for your particular situation.

1. **Does glaucoma always occur in both eyes?**

- **Answer:**
- The most common type of glaucoma (open-angle glaucoma) often occurs in both eyes but not always. Often it is worse in one eye. Some types of glaucoma result from other disease—like uveitis, or from injury—and these can occur in one eye.

1. **Is there any way to lower eye pressure using home remedies?**

- **Answer:**
- Eating a healthy diet can maintain your overall health and may be good for your eye health as well. Regular exercise has been shown to reduce the eye pressure in some individuals with open angle glaucoma. Many herbal remedies are advertised for glaucoma but none have been proven effective in treating this potentially blinding disease. Herbal supplements should never be used in place of proven therapies that are prescribed by your doctor. You should always discuss any of these types of treatments with your ophthalmologist before trying them.

1. **What causes the foreign body sensation in acute angle closure?**

- **Answer:**
- When the IOP (intraocular pressure) rises during an acute angle closure attack (when the drainage angle inside eye is completely blocked due to angle-closure glaucoma), there can often be a great deal of discomfort, but patients feel that discomfort in a variety of ways. Some patients have a headache or brow ache, others have a deep, penetrating pain behind or around the eye, and others have a foreign body sensation. Foreign body sensation is usually caused by corneal edema (swelling of the cornea with fluid) which makes the corneal surface uneven. If you have these or other concerning symptoms, see your ophthalmologist as soon as possible.

1. **Does caffeine make glaucoma worse?**

- **Answer:**
- There may be a small effect of increased intraocular pressure with caffeine consumption. However, there is no evidence that this effect is clinically significant. I would say go ahead and enjoy your coffee, tea or energy drink—always with an “eye” toward reasonable moderation, as in all things.

1. **Does severe arcus senilis cause glaucoma?**

- **Answer:**
- Arcus senilis—also known as corneal arcus—is caused by deposits of cholesterol in the outer ring of the cornea. It often can appear as a blueish, white ring in people older than 60. This is a normal deposition of cholesterol and is not a sign of any eye disease unless it is only in one eye, or occurs at a young age. While arcus can have a minimal effect on eye pressure, it has no relationship to the formation of glaucoma.

1. **I have open-angle glaucoma. Can I safely take Imodium occasionally to control my IBS?**

- **Answer:** It is safe. Imodium should not affect your open-angle glaucoma at all.

1. **Will my eye pressure go higher than it was before treatment if I stop taking my glaucoma medication?**

- **Answer:**
- Timolol is a beta blocker which lowers eye pressure by decreasing aqueous production (fluid inside the eye that helps the eye keep its shape). This effect has a fairly rapid onset (within 20 minutes) and can last for up to 24 hours after a single dose. There is no evidence with any of the major classes of anti-glaucoma medications that the pressure after stopping the medication will go higher than the pre-treatment pressure. It is always best to have a conversation with your ophthalmologist before stopping any glaucoma medications, unless you are having a severe reaction to the medication.

1. **Can you tell me what treatment is recommended for pseudoexfoliation glaucoma?**

- **Answer:**
- Similar to other types of glaucoma, the goal with pseudoexfoliation glaucoma (when flaky dandruff-like deposits inside the eye block fluid from leaving the eye, causing pressure to rise) is to reduce eye pressure to prevent additional damage to the optic nerve.
- Typically, we first try topical eye drops to reduce eye pressure. Patients with pseudoexfoliation glaucoma that is mild to moderate also tend to respond particularly well to selective laser trabeculoplasty, which is a low-risk, in-office procedure.
- There are also some minimally invasive glaucoma surgery options for mild to moderate pseudoexfoliation glaucoma, such as iStent, trabectome, or endoscopic cyclophotocoagulation. If the glaucoma is more advanced or the patient needs significant eye pressure lowering, more traditional glaucoma surgery such as trabeculectomy or aqueous shunt implant is recommended.
- The best thing a patient with pseudoexfoliation glaucoma can do is to live a generally healthy lifestyle with good diet and exercise, be diligent about following their ophthalmologist's instructions for using medications, and returning for regular follow-up with their ophthalmologist so any changes in the glaucoma or eye pressure can be noted right away.
- Finally, it is important to note that some patients with pseudoexfoliation also have narrow angles (when the colored part of your eye is pushed too far forward), which can put you at risk for angle closure attacks of elevated eye pressure. Everyone with pseudoexfoliation should be evaluated by their ophthalmologist for narrow angle.

1. **Does famotidine contribute to glaucoma?**

- **Answer:**
- Famotidine can be safely used with glaucoma and is not known to cause glaucoma.

1. **I have undergone an Ahmad’s implant procedure to treat my glaucoma. How soon after Ahmed implant surgery can I go swimming?**

- **Answer:**
- The safest strategy is to avoid swimming until your surgeon declares healing to be complete and you no longer need eye drops.

1. **Is it safe to exercise and wear contacts after a trabeculectomy?**

- **Answer:**
- Most exercise is fine after a trabeculectomy, once your vision has recovered. You must be very careful to protect your eyes with safety glasses for sports or tool usage—specially gardening (avoiding branches), playing racquetball, tennis, squash, and baseball, and using power tools.
- Swimming is not recommended after a trabeculotomy. It is best to avoid getting any water in your eyes while showering or bathing to avoid the normally harmless bacteria (which normally is in the water we drink and bathe in) from infecting your eyes.
- The bad news: you should not wear soft contacts after a trabeculectomy. Soft lenses increase the risk of infection inside the eye after trabeculectomy by a factor of four, and the results of the infection inside the eye can be devastating. Soft lenses can irritate or rupture the filtration "bleb" that is created during the trabeculotomy procedure. This can cause infection which can lead to loss of vision, loss of the anti-glaucoma function through scarring, or even loss of your eye from endophthalmitis or internal infection of the eye.
- If you can't get adequate vision with glasses, your ophthalmologist might, in some cases, fit you with rigid gas permeable contacts—but check first with your glaucoma specialist.

1. **Does prolonged steroid use affect vision?**

- **Answer:**
- Yes, steroids can cause both glaucoma and cataracts. They should be used judiciously. If you’ve been on steroids for longer than several weeks, you need to be monitored by an ophthalmologist.

1. **How safe is corneal transplantation for someone who has glaucoma?**

- **Answer:**
- Many patients with glaucoma undergo successful corneal transplantation surgery. However, the risk of graft failure is increased when their pressure does not stay in good control. Patients may need additional glaucoma therapy after surgery since often a slight increase in their intraocular pressure is noted. All patients need to be closely monitored to ensure that their transplant remains healthy.

1. **Can I take benzodiazepines (sedatives) if I suffer from glaucoma?**

- **Answer:**
- Benzodiazepines can dilate the pupil, so it is conceivable that they could cause problems in a patient with narrow-angle glaucoma. For patients with open-angle glaucoma, the use of benzodiazepines should not present a problem. Please check with your ophthalmologist if you are unsure what type of glaucoma you have.

**Cataract / 10**

1. **Can I wear contact lenses after cataract surgery?**

- **Answer:**
- To clarify some of these terms for our readers, it sounds as though you had cataract surgery with implantation of monofocal lenses in each eye. These monofocal lenses were used to achieve monovision, where one eye was set for distance and the other set for near.
- To answer your question, unless you have another ailment, you can almost certainly wear contact lenses after having cataract surgery, and there are a variety of choices are available to you. You can wear a contact that improves the distance vision in the near eye, you can wear a contact that improves the near vision in the distance eye, or you could even wear a contact to enhance the near vision in the near eye.

1. **Is it safe to have an MRI after cataract surgery?**

- **Answer:**
- Yes, it's safe. However, any interior magnetic items (such as a pacemaker or inner ear implant) should always be discussed with your doctor before an MRI.

1. **Is it safe to have YAG laser with a PVD (posterior vitreous detachment) ?**

- **Answer:**
- The YAG laser used during a posterior capsulotomy poses no risk to the vitreous inside the eye or its age-related detachment process if already underway. However, YAG capsulotomy does carry a low but real risk of retinal tear (average risk is less than 1%) and this risk is greatest if the PVD (posterior vitreous detachment) is in process or has recently occurred.
- Based on recent research it is typically recommended that you wait at least 6 months following a complete PVD before proceeding with YAG capsulotomy to decrease the risk of retinal tear and detachment. There may be special circumstances affecting the timing of your procedure so please discuss this question with your eye surgeon.

1. **Will radiation treatment affect my Intraocular Lens (IOLs)?**

- **Answer:**
- I'm sorry you're dealing with these problems. You can be rest assured that there is no evidence radiation treatment will affect your intraocular lenses.

1. **Is pupil size a factor in lens choice for cataract surgery?**

- **Answer:**
- Pupil size may be a consideration when choosing certain types of intraocular lenses (IOLs) before cataract surgery. The Light Adjustable Lens (LAL) requires at least a 7 mm dilated pupil, so the power can be adjusted after surgical healing. Pupil size can also affect the performance of multifocal IOLs. A too small pupil may decrease the sharpness of near and intermediate vision, while a bigger pupil may cause problems with glare and distance vision.

1. **Is it possible to have two different types of cataracts?**

- **Answer:**
- Yes it’s possible and not uncommon. Cataracts differ in appearance and where they form on the eye’s lens. People can have a combination of cataract types while others will only have one.

1. **What are the risks of not removing a cataract?**

- **Answer:**
- The main risk of not having a cataract removed is that you will be living with compromised vision from the cataract that is known to result in a higher incidence of falls as well as other problems. If your vision is not bothersome and you feel you can get around and do all activities of daily living, there is no harm in waiting. It is possible to wait too long, making the cataract operation more difficult if the cataract becomes mature or very dense.

1. **Can cataract surgery trigger NAION (non-arteritic anterior ischemic optic neuropathy)?**

- **Answer:**
- This is an excellent question without a clear answer. Research studies suggest that there is a slightly higher risk of NAION (non-arteritic anterior ischemic optic neuropathy) after cataract surgery. This risk may be higher in patients with a history of NAION in their other eye. There are other rare complications from cataract surgery that cause permanent vision loss including infection inside the eye (endophthalmitis) and retinal detachment. Because cataracts slowly worsen over time, but do not typically damage the eye otherwise, patients are encouraged to weigh the risk of these uncommon complications against the relative interference of visual function from the cataract**.**

1. **Can a cataract cause monocular diplopia?**

- **Answer:**
- Monocular diplopia is seeing two images (either separate or overlapping or with ghosting or shadowing) from one eye with the other eye covered. In your case, you state that you have this in both eyes, meaning that with each eye covered the uncovered eye is having these symptoms. Cataracts can definitely cause this. As can corneal disease, severe dry eye and many other conditions.

1. **Will cataract surgery repair my worsening nearsightedness?**

- **Answer:**
- After macular hole surgery, cataract formation and worsening of cataract are common. As the cataract forms, it generally induces more nearsightedness and that can be corrected with cataract surgery.

**[Infections] , [Conjunctivitis], and [dry eye] / 15**

1. **Are styes in the eye contagious?**

- **Answer:**

Styes are not contagious, but rather a local infection or inflammation of the oil-producing glands of the eyelids. Local treatment is usually warm compresses and most will resolve without antibiotics. Few will progress to significant eyelid infections marked by substantial swelling and pain. They may be recurrent in some people, possibly because of eyelid chemistry specific to a patient.

1. **Can the fungi from athlete’s foot be transferred to the eye?**

- **Answer:**

Athlete’s foot is caused by the fungus Tinea, or ringworm. It is typically spread by direct contact and burrows in dead, keratinized skin cells. Since the eye is covered with non-keratinized cells (similar to the inside of the mouth), ringworm does not infect these regions. However, ringworm can cause an infection of the surrounding eyelid skin. The surface of the eye is susceptible to other fungal infections such as Candida.

1. **Can sinus infections cause puffy eyelids?**

- **Answer:** Yes. A sinus infection as well as allergies, thyroid eye disease (Graves’), long-term steroid use, renal failure, age and genetics, etc., can cause puffy lids. Please see an ophthalmologist to help find the cause of your puffy eyelids.

1. **Can you wear contacts with toxoplasmosis?**

- **Answer:**

The prior diagnosis of ocular toxoplasmosis will not impair your ability to switch to contact lenses. The contact is placed at the front of the eye so it would not affect any existing toxoplasmosis scars in the retina at the back of the eye. Be sure to clean and handle your contact lenses as instructed by the manufacturer and your prescribing eye care professional.

1. **What are the signs that corneal keratitis is healing?**

- **Answer:**
- Corneal keratitis (painful inflammation of the clear, dome-shaped window at the front of the eye) applies to a wide group of disorders each with different causes and treatments. However, in the most common form (epithelial keratitis), on the outermost layer of the cornea, patients feel a foreign body sensation (feeling like something is in your eye), especially when blinking. Depending on what layer of the cornea is affected, other common symptoms include eye redness, pain, light sensitivity, and hazy vision. Signs to the patient that they are healing are diminishment of their symptoms. Similarly, from the ophthalmologist's perspective, we look for improvement of the corneal inflammation, until it appears back to normal.

1. **If I get conjunctivitis once can it come back?**

- **Answer:**
- Conjunctivitis is inflammation of the conjunctiva, which is the thin transparent membrane that covers the white part of the eye (sclera). Different types of conjunctivitis can occur. Allergic conjunctivitis is caused by allergens (pollen, dust, pets or perfumes) contacting and irritating the conjunctiva. This type may be the most common and it is not contagious (catching or spreading) and has a tendency to recur. Two other types of conjunctivitis, bacterial and viral, are highly contagious. Bacterial conjunctivitis refers to an infection caused by bacteria attacking the conjunctival layers. Viral conjunctivitis refers to viruses that can infect the conjunctiva. If the person comes into contact with the bacteria or virus again, they could get conjunctivitis again. Herpes virus can cause recurrent conjunctivitis (like recurrent cold sores). Chemical conjunctivitis refers to chemicals or thermal injuries affecting the conjunctiva layers.

1. **Can a corneal infection affect my brain?**

- **Answer:**
- If the infection stays confined to the cornea, it cannot spread to the brain. If the infection spreads into the back portion of the eye, this is referred to as endophthalmitis. In rare instances, an infection in the eye can spread to the brain if it has caused endophthalmitis; however, this is very rare. A specialist should be able to keep the infection confined to the front of the eye with topical and/or systemic antibiotics depending on what has caused the infection based on cultures of the cornea.

1. **Can the conjunctivitis adversely affect the pressure in my eye?**

- **Answer:**
- Generally an upper respiratory tract infection associated with a viral conjunctivitis does not affect the eye pressure. In some circumstances, if the eye becomes extremely congested, the eye pressure could increase a small amount, but this would not be typical. If you have concerns about the nature of your eye, it is important to see your ophthalmologist for a full evaluation.

1. **Along with eye drops, will sunlight help pink eye?**

- **Answer:**
- The term “pink eye” usually refers to an eye infection caused by a virus or bacteria, though sometimes it can have other causes, such as allergies. Regardless of the cause, there is no evidence that direct exposure to sunlight will help pink eye, and too much sun can be bad for your eyes, just like your skin.
- In addition to your prescription eye drops for pink eye, we recommend washing your hands often and using cool compresses (a clean, cold damp washcloth laid over your closed eyes) as needed for comfort. If your symptoms don’t improve then you should let your eye care provider know.

1. **Can CMV be reactivated in an HIV positive person?**

- **Answer:**
- CMV can be reactivated in an HIV+ person, mostly when their immune competence deteriorates. This would not be related to episodes of conjunctivitis, but rather it would be the result of breakdown of their overall ability to mount an effective response to the CMV virus.

1. **My daughter has pink eye. How long is pink eye contagious and when can I send her back to school?**

- **Answer:**
- Pink eye (conjunctivitis) generally remains contagious as long as the eye continues to tear and produce a discharge. Bacterial conjunctivitis can last up to 10 days, and shorter if an antibiotic drop is administered. In general the patient can return to school or day care as soon as the symptoms of redness and discharge are greatly improved. Viral conjunctivitis can last for up to 14 days. There is no curative treatment for viral conjunctivitis. It is appropriate for a child to return to school or child care when tearing and discharge are greatly improved.

1. **How can I clear up conjunctivitis due to an allergic reaction from eye ointment?**

- **Answer:**
- Allergic conjunctivitis is much like any other allergic condition. A person can be allergic to a wide variety of substances. The treatment of allergic conjunctivitis is most successful by removing the entity that is stimulating the allergy. Medications, both oral and topical, can be used to suppress the allergic reaction. I suggest you consult your ophthalmologist for a conversation more specific to your condition.

1. **Can dry eyes cause double vision?**

- **Answer:**
- Yes. Double vision can happen when the brain gets two different images either from each eye or from within the same eye. While double vision is not the most common symptom of dry eye syndrome, the drier the eyes are, the fuzzier the image is. Sometimes this happens when a patient has subtle eye misalignment, but with the additional blur from dry eyes, now they notice their double vision. This can also be worsened with fatigue, which can affect the eye muscles.

1. **Should I take out my contacts to apply artificial tears?** **or is it OK to wear my contacts?**

- **Answer:**
- Yes, you can use the eye drops with your contacts still in. Preservative-free artificial tears are safe to use with contact lenses, as well as lubricating drops made specifically for contact lens wearers (as labeled). Many artificial tears use preservatives which can adhere to your contact lens causing irritation and may affect your eye health and comfort. If you use drops more than four times per day, preservative-free drops may be a better option. Ask your your ophthalmologist which drop is best for you.

1. **Can my daughter use eye drops for dry eyes after swimming?**

- **Answer:**
- Lubricating eye drops are safe in children. Typically, the artificial tears are applied up to six times a day for comfort. If drops are needed more often, a preservative-free artificial tear is better as it has less risk for irritation than drops with preservatives. I would advise you to avoid using a drop advertised as getting the red out as this type of drop can result in "rebound" redness when the drop wears off. If the eye irritation persists, it would be best to have your child examined by an ophthalmologist to make sure there is not another cause for their eye problems.

**Astigmatism : [Glasses, Contact lenses, and Vision correction], [Keratoconus] / 19**

1. **Can glasses fully correct my astigmatism?**

- **Answer:**
- There are two types of astigmatism (when the eye is more oval-shaped than round): regular and irregular. Glasses or soft contacts can usually correct regular astigmatism to 20/20, but if it is a high degree of astigmatism, the eye may not be correctable to 20/20.
- Glasses or soft contacts cannot usually correct irregular astigmatism completely, but customized contacts—such as rigid gas permeable (RGP) or scleral contact lenses—can usually overcome this problem and may be able to get the eye back to 20/20.
- If you are unhappy with your vision using glasses, the next step is to consider custom-fit contact lenses.

1. **Can a 5-year-old have astigmatism with no symptoms?**

- **Answer:**
- It is possible to be asymptomatic with a moderate amount of astigmatism. However, I would expect the vision to be reduced if measured on an eye chart or to see the child squinting while reading the chart. Because uncorrected astigmatism can lead to amblyopia (sometimes called “lazy eye”), be sure an ophthalmologist monitors your child’s developing vision and gets the right treatment if needed.

1. **What is the difference between against-the-rule and with-the-rule astigmatism?**

- **Answer:**
- Astigmatism is when your eye’s cornea or lens is irregularly shaped. Normally, the cornea and lens are curved equally in all directions.
- If we view the eye as a sphere, “against-the-rule” is astigmatism where the steepest curve lies near the 180-degree meridian (imaginary line connecting east and west points of the cornea), and “with-the-rule” astigmatism (line connecting north and south points) is near the 90-degree meridian. With-the-rule is by far the most common type of astigmatism.

1. **If I have astigmatism in one eye, do you always have it in the other eye?**

- **Answer:**
- Two forms of astigmatism exist. Regular astigmatism is a type that is corrected by glasses or soft contact lenses, and irregular astigmatism is typically created by various eye conditions or eye diseases and is corrected by hard contact lenses or surgical options. While astigmatism is most often bilateral (both eyes) that is not always the case. In addition, astigmatism is rarely exactly the same amount in both eyes. Some eye conditions can lead to asymmetrical amounts of astigmatism between two eyes. Even something as simple as a large stye (hordeolum) or chalazion (blocked oil gland in the eyelid) can push on the eye and induce temporary astigmatism in one eye from pressure on the wall of the eye that distorts the cornea shape.

1. **Does astigmatism-correcting cataract surgery extend the recovery process?**

- **Answer:**
- While a normal cornea has the shape of a slightly peaked dome, similar to the smaller end of an egg, we often talk about the cornea as a round sphere like a basketball. Astigmatism is the name we give to a cornea that looks more like a football than a basketball. For example, if you picture a football lying on a clock face with the long axis running from 3:00 to 9:00, the curvature is steeper in the axis at 12:00 on the clock face and flatter in the axis at 3:00, 90 degrees away. This is called “regular” astigmatism.
- This difference in the curvature produces two or more focus points on the retina which creates blurred vision. Eyeglasses and contact lenses are used to correct astigmatism and can produce excellent vision. There are times when a person might wish to correct his/her astigmatism with surgery to remove this astigmatism and it is common to do this at the time of cataract surgery, either with a toric implant lens or with limbal reading incisions. However, if the axis of astigmatism does not line up to make a perfect cross (perpendicular lines) then it is called “irregular” astigmatism and may require a hard contact lens or corneal surgery to correct.
- Limbal relaxing incisions correct regular astigmatism by flattening the overly steep curves in the cornea. The ophthalmologist can make these incisions by hand with a tiny blade or with a laser. Since relaxing incisions are an elective procedure, most insurance programs consider them to be cosmetic in nature and do not cover the added cost of this procedure.
- There are really two different aspects to recovering after cataract surgery: one that deals with the healing of the eye and the other deals with the final clearness of vision. The “recovery” from surgery is not prolonged with the relaxing incisions. There can be a mild scratchy feeling for a few days, but this seldom causes any recovery issue. The final visual sharpness you will have after cataract surgery can take a bit longer with astigmatism-correcting incisions. The cornea takes a little more time to stabilize after relaxing incisions, but this usually happens within a few weeks, depending on the degree of astigmatism.

1. **Can cataracts cause astigmatism?**

- **Answer:**
- Yes. As the lens thickens and changes shape while developing a cataract there can be induced astigmatism due to these changes.

1. **Could contact lenses cause redness around the iris?**

- **Answer:**
- Symptoms of eye redness, irritation, blurred vision, or pain are never normal. Contacts should feel comfortable with good vision and no redness. The red ring may be a side effect of a contact lens which is damaged (torn, or has protein on it) or fitting too tightly. It can also be a reaction to the contact lens cleaning solution or the contact lens itself.
- Then again it may be that the contact lenses aren't the culprit at all, and the redness may be secondary to 1) a bacterial or viral eye infection (conjunctivitis or corneal ulcer); or 2) an inflammation in the eye called iritis, which can be idiopathic (arising from an unknown cause). It could also be a sign of juvenile rheumatoid arthritis or ankylosing spondylitis, or an allergic reaction to pets, pollen, or other environmental factors.
- In any case, have your son wear glasses now and make an appointment with your ophthalmologist. Be sure to bring the offending contacts and contact lens cleaning solution with you to the appointment. Recently one of my patients was using a non-compatible contact lens cleaner and was putting diluted hydrogen peroxide in his eye. I asked him why and he said "all contact lens cleaners are the same." This is incorrect and some solutions will irritate your eyes. Your doctor will inspect your son's eyes, contacts and solution and then, if everything looks fine, put the contacts in and see how they fit.

1. **I'm 62 years old and my distance vision has improved without spectacles. Why is that?**

- **Answer:**
- Some call this "second sight" which has a simple physiological explanation. As the lens of the eye hardens as we age (the predecessor of frank cataracts) it changes the way light is "bent" as it enters the eye much the way different prescriptions in a pair of glasses do. So if you were hyperopic (needed plus correction) and you start developing pre-cataract changes, your vision may temporarily go through a phase where either the near or distance vision gets better. Unfortunately as you continue to age, this process will make your overall vision worse and you will need cataract surgery. Enjoy it while you have it!

1. **Is it OK to wear my contact lens with a swollen retina?**

- **Answer:**
- If the cornea and the front of the eye is not injured, then it is fine to wear a contact lens, as the retina is in the back of the eye.

1. **Is my iPhone usage linked to my deteriorating near vision?**

- **Answer:**
- It is likely that your decrease in vision is related to being a bit older and losing some of your natural accommodative (focusing) capacity. Try using a pair of readers at night time and having a good light source. Of course, a complete eye examination by an ophthalmologist to rule out glaucoma and other issues that can affect your sight as you age is important.

1. **Do scratched eyeglasses have to be replaced?**

- **Answer:**
- Looking through a scratched lens may be annoying and could distract your eye’s gaze, but it won’t harm the eye’s optical system. It is possible that the scratch(es) could be distracting enough to cause eye strain and headaches. Some may wish to replace their scratched lenses/glasses, but there’s no long-term harm in continuing to use them.

1. **Why Do My Eyes Burn After Inserting My Contacts?**

- Answer:
- Redness and burning of the eyes after inserting contact lenses can be due to physical irritation from lenses that do not fit properly or are damaged, allergy to chemicals in the cleaning and storing solutions, sensitivity to broken down tear protein deposits in the lens, swelling of the cornea (the clear window on the front of the eye), or an infection of the eye. Do not force the use of the contact lenses. Wear your backup glasses, leave the contact lenses out, and see an ophthalmologist promptly!

1. **I am a 51-year-old female with type 2 diabetes. Would an intraocular lens or a corneal inlay work better to treat presbyopia?**

- **Answer:**
- At your age, a Symfony or multifocal lens would give the longest-term gain—assuming that the eye is healthy enough for such an implant. The corneal inlay would give you near vision for the short term but you would lose a good amount of vision in that eye during dark hours such as driving at night. See your ophthalmologist for a fuller discussion about which option would work best for you.

1. **Can wearing a stronger eyeglass lens than necessary make your vision worse?**

- **Original question :** I recently had an eye exam. The technician checked the prescription of my glasses, and both lenses were -5.25. I checked the prescription my eye doctor gave me 3 years ago. The prescription for my right eye was -5.00 and my left eye was -5.25. Therefore, the right lens of my glasses was raised .25 higher than it should have been. Would wearing a stronger lens make my eye worse and cause the prescription of my eye to go up?
- **Answer :** These changes are not significant, and there is no reason for concern.

1. **Can you use contact lens rewetting drops to moisten your bare eye?**

- **Answer:**
- You can use an unopened contact lens rewetting solution in your eyes as an alternative to over-the-counter ordinary lubricating eye drops if needed. They are safe for your eyes. However, never use contact lens rewetting solution that has been opened and used by someone else.

1. **Is Keratoconus a Refractive Error?**

- **Answer:**
- Keratoconus is a disease of the cornea (the clear front part of the eyeball) in which the cornea becomes abnormally thin and irregular in shape. This can result in myopia (nearsightedness) and/or astigmatism (optical irregularity.) Myopia and astigmatism are both refractive errors of the eye; but in many patients with keratoconus, it is not possible to correct these refractive errors adequately with eyeglasses, so many patients with keratoconus must wear contact lenses--usually hard, gas-permeable contact lenses--to achieve clear vision.

1. **Can age and eyelid drooping interfere with hybrid lenses used for keratoconus?** **Is aging causing my discomfort from contact lenses?**

- **Answer:**
- Hybrid contact lenses combine the visual sharpness of a rigid gas permeable (RGP) contact lens with the comfort of a soft lens. The comfort of hybrid or any other type of contact lens is related to the health of the eye. While eyelid height and age do not directly affect the comfort of the contact lenses, there are other factors of the eyelids that can impact tolerance. Your eye doctor will focus on whether or not your eyes are lubricated enough for contact lenses or if the underside of the eyelids are swollen and inflamed in response to the contacts (giant papillary conjunctivitis). They will also examine the fit of the contact lens on the surface of the eye, as a poorly fit contact lens can increase discomfort throughout the day.

1. **How effective is corneal collagen cross-linking for keratoconus?**

- **Answer:**
- Keratoconus is a disease of the cornea that leads to thinning and distortion of the cornea and, often, reduced vision. Often the condition can be treated with glasses or contacts, but some cases require a corneal transplant. Recently, a new treatment for keratoconus, called collagen cross-linking, became available abroad in Europe and in this country, at least on a somewhat limited basis. The treatment consists of applying a specific vitamin (riboflavin) as a drop to the cornea during treatment and using ultraviolet light to induce the cross-linking. Corneal cross-linking does show promise for stopping or at least slowing the progression of the disease in certain cases at certain stages. Unfortunately, it is not fully approved in the United States as of this writing. It is also not universally available as it is only being performed in a clinical research setting, and may be quite expensive. The treatment does appear to be effective when properly prescribed. Risks for the procedure are few but do exist. Ask your ophthalmologist if this treatment is right for you, as it just may be effective and helpful.

1. **Can one be born with Keratoconus?**

**Answer:**

Patients aren't born with keratoconus. Rather, they start showing the first signs of keratoconus around the age of puberty when the cornea naturally begins to thin and protrude from the rest of the eyeball. While researchers have found a higher incidence of keratoconus amongst related individuals, there have not been any genes isolated for the disease. The current thinking is that keratoconus is due to a combination of factors related to having an excessively stretchable cornea and eye rubbing, which causes the cornea to warp into an abnormal shape. In certain genetic diseases such as Marfan's syndrome or Ehlers-Danlos syndrome, patients have more stretchable tissue. Patients with severe Atopic Disease or Down's Syndrome tend to rub their eyes frequently and vigorously. In all of these conditions, there is a higher risk for developing keratoconus.

**Diabetic Retinopathy / 8**

1. **Is it possible to reverse mild diabetic retinopathy?**

- **Answer:**
- Diabetic retinopathy is when high blood sugars damage blood vessels in the retina. When blood sugars and other conditions like blood pressure are well-controlled, diabetic retinopathy can improve and even resolve over time.

1. **How long should you avoid swimming after an anti-VEGF injection?**

- **Answer:**
- It is recommended that no water enter the eye for 48 to 72 hours following an anti-VEGF injection. However, it is best to follow your ophthalmologist’s specific recommendations as they may vary.

1. **Is a multifocal IOL safe for use if you have uncomplicated diabetes?**

- **Answer:**
- Yes, as long as you have no diabetic retinopathy. Be sure to confirm with your ophthalmologist after you have had a complete dilated eye exam that the diabetes has not affected your vision.

1. **Is Avastin a good treatment for diabetic macular edema?**

- **Answer:**
- Intravitreal injections of Avastin or similar drugs have been proven to be the best available treatment for diabetic macular edema. A recent study demonstrated that Avastin works as well as other more expensive drugs when the vision is relatively good. However, when the vision is approximately 20/50 or worse, another drug called Eylea may be a better choice. You should discuss your level of vision with your doctor and ask about other drugs.

1. **What eye symptoms should I look for with a pre-diabetes diagnosis?**

- **Answer:**
- Usually, pre-diabetes has no effect on vision. Often pre-diabetes can be eliminated through change in diet and weight loss.

1. **Can Diabetes Cause Eye Floaters?**

- **Answer:**
- Diabetes—a disease in which the body doesn’t process sugar correctly—will not cause floaters by itself. However, if you develop the more advanced form of diabetic eye disease, proliferative diabetic retinopathy, you are at risk of bleeding in the eye which will cause floaters.

1. **How Soon Can Diabetic Retinopathy Develop in a patient with type 1 diabetes?**

- **Answer :** It typically requires a minimum of five years, and often longer, before any signs of diabetic retinopathy develop. The current recommendations for people with type 1 diabetes suggest a complete eye examination at five years after diagnosis. Furthermore, diabetic retinopathy is painless so if the pain continues she should see an ophthalmologist for a complete eye examination.

1. **What procedure is used for removing blood from the back of the eye?**

- **Answer:**
- The procedure for removing blood from the vitreous cavity of the eye is called a vitrectomy. When blood enters this area due to diabetes, trauma, injury, retinal tears, retinal detachments, vascular occlusions, or intraocular tumors, it can obstruct vision, depending upon the amount and location of the blood. This operation must be performed by an ophthalmologist. Some general ophthalmologists are very skilled at this, but most are performed by fellowship-trained vitreoretinal surgeons. Some call them retinologists, but I personally do not like the term.

**Macular Degeneration / 6**

1. **How successful is laser for dry age-related macular degeneration (AMD)?**

- **Answer:**
- The research performed to date suggests that in the long-term, laser treatment does not significantly decrease the rate of progression or severity of dry age-related macular degeneration (AMD). There is ongoing research into several treatments for dry AMD, which will hopefully provide us with a new treatment for dry AMD in the near future.

1. **Is multiple sclerosis associated with AMD or uveitis?**

- **Answer:**
- While MS has not been associated with macular degeneration (or AMD, age-related macular degeneration), it can be associated with uveitis. In fact, recent studies have suggested that uveitis is significantly more common among those with MS versus the general population, although the precise incidence and prevalence varies.

1. **Do wavy lines on an Amsler grid always mean you have AMD?**

- **Answer:**
- Wavy lines on an Amsler grid do not always mean you have age-related macular degeneration, or AMD. A cyst or macular pucker can also cause waviness.
- The Amsler grid is used to monitor many conditions affecting the macula (part of the retina used for crystal clear central vision) and optic nerve. Other eye conditions, like dry eye, can also cause the lines to appear wavy. When the lines appear wavy or distorted on the Amsler grid you should see your ophthalmologist.

1. **Does macular edema mean your dry AMD progressed to wet AMD?**

- **Answer:**
- Macular edema means that there is fluid in or swelling of the retina. One of the causes of macular edema is wet macular degeneration, but there are a number of other potential conditions that could lead to this finding. Your ophthalmologist or retinal specialist will be able to explain what your particular finding means.

1. **Should I get an OCT scan to monitor early AMD?**

- **Answer:**
- In general, optical coherence tomography (OCT) has not been shown to play a critical role in monitoring progression of early AMD. Rather, you should obtain retinal photographs so your eye doctor can determine any changes from baseline. Should you develop symptoms of wavy or distorted vision, OCT can be valuable in identifying fluid under the retina that would suggest progression to a later stage of AMD, or what is called neovascular or wet AMD.

1. **Does cataract surgery accelerate the progression of dry AMD ?**

- **Answer :** the best available evidence suggests that cataract surgery does not accelerate the progression of dry AMD.

**Retinal Detachment / 12**

1. **Why am I still seeing wavy lines after vitrectomy? I had a pucker and almost a blind spot. I notice that the blind spot or blurriness is gone but I still have wavy vision .Will it improve with time?**

- **Answer:**
- One week is way too soon to be judging the success of your vitrectomy. The fact that you already notice improvement in the blind spot and blurriness is remarkable and should be cause for great optimism. The fact that you still have wavy vision is typical. The epiretinal membrane caused distortion of the retina cells, which leads to waviness of vision. Over the next few months, there can be significant realignment of the retinal cells with accompanying improvement in your symptomatic waviness. However, in most cases, there still are some persistent changes in your vision.

1. **What does a sudden onset of an arc of slashing light on the periphery of one eye indicate?**

- **Answer:**
- It is urgent and you need to see your ophthalmologist very soon. The inside of an eye is filled with a thick, clear jelly called the vitreous humor. As we age, the vitreous humor loses some of its water content and shrinks. At that point it is no longer big enough to fill the entire inside of the eye and it pulls away from the retina. As it pulls away, it often tugs on the retina and that causes an arc-like flash of light in the periphery of the vision. A small number of people who undergo this normal process will develop a tear in the retina or a detachment of the retina. This can be vision threatening and could result in blindness if not treated soon enough. Anyone with the symptoms of flashes of light with or without new black spots (new vitreous floaters) in their vision must be examined by the ophthalmologist right away.

1. **Can I swim in the ocean two months after torn retina repair?**

- **Answer:**
- After two months, your laser scarring around the retinal tear should be mature. There is no harm for you to be swimming, whether in freshwater or salt water.

1. **Can I wear my contact lenses after laser retinal surgery?**

- **Answer:**
- There are generally no restrictions following retinal laser and contact lens wear the next day should be fine. But be sure to confirm with your surgeon as there may be considerations unique to your situation**.**

1. **How long will the gas bubble stay in my eye after retinal detachment treatment?**

- **Answer:**
- Gas bubbles and laser can be used to repair retinal detachments. You should ask your retinal surgeon what type of gas was used and how long he or she anticipates it being present for. There are typically two types of gases that we use. One is called SF6 and lasts about two weeks, and the other is called C3F8 and lasts about six to eight weeks.

1. **Can my retinal thinning be a sign of cognitive decline?**

- **Answer:**
- Retinal thinning can mean many things. If the thinning is in the peripheral retina, it can be from a condition called lattice degeneration, which is associated with an increased risk of retinal tears and retinal detachment. Central retinal thinning can be associated with a number of conditions including myopia, glaucoma, and age-related macular degeneration.
- There have been two recent studies that have suggested a correlation between thinning of one of the retinal layers (the retinal nerve fiber layer) with lower performance on cognitive testing. However, the percent of patients who went on to develop cognitive decline was very small and this finding has not been recommended as a screening test for dementia.

1. **Can straining, coughing or vomiting lead to retinal hemorrhage or detachment?**

- **Answer:**
- Straining, coughing or vomiting can lead to retinal hemorrhage but not a posterior vitreous detachment. Straining, coughing or vomiting do not lead to a retinal tear or detachment. Ask your doctor to recommend the best way to help relieve your constipation.

1. **Will an accidental rubbing of my eye affect my recently repaired retina?**

- **Answer:**
- Rubbing your eye will have no damaging effect on the benefits of the laser treatment for your small retinal tear.

1. **Can pigmented epithelium detachment (PED) lead to retinal detachment?**

- **Answer:**
- PED (when a layer of cells under the retina no longer lies flat against the back of the eye as it should) does not on its own lead to retinal detachment. When this layer of cells (called the pigment epithelial layer) separates from the back of the eye and elevates, the retinal layer, above it, can become raised as well. This typically does not lead to retinal detachment. However, sometimes abnormal blood vessels can grow under the retina (choroidal neovascularization) with PED. This type of PED can be linked to retinal detachment that happens in the macula, in the middle of the retina.

1. **Is it possible to have a retinal tear without any symptoms?**

- **Answer:**
- Yes, you definitely can develop a retinal tear without any symptoms. While most retinal tears are associated with the sudden onset of flashing lights and floaters, symptoms can vary widely between patients, including having no symptoms whatsoever.

1. **Can alcohol affect my eye after having a retinal detachment surgery?**

- **Answer:**
- Alcohol should not have any effect on your eye after retinal detachment surgery.

1. **Can I get a lens implant if I've had a retinal detachment surgery?**

- **Answer:**
- Yes. Many people who have had surgery for a detached retina will then develop cataracts. These people should definitely have an intraocular lens at the time of cataract surgery.

**Floaters and Flashes / 10**

1. **How do I differentiate between a retinal tear and an ocular migraine?**

- **Answer:**
- Flashing lights (photopsias) can happen with both retinal tears and migraines. Lights seen with a retinal tear tend to come and go with no defined length of time. They are usually brief flashes and do not affect vision unless they happen with floaters as well. Retinal tears happen in a specific eye, so the flashes of light only appear in one eye. With migraine, flashes usually last up to 30 minutes. The flashes may grow from smaller to larger and appear in both eyes. You may see a blank spot that diminishes your vision and improves as the flashes resolve. These flashes may be jagged and geometric and happen in rhythm with your pulse. Flashes from migraine usually don’t happen with floaters in your vision as well. You may or may not have a headache or nausea after the flashes.
- Although this is how retinal tear and migraine usually appear, it can sometimes be difficult to determine the cause of flashes. A complete eye exam is needed to find out what is causing your symptoms.

1. **Does drinking water eliminate flashes?**

- **Answer:**
- The vitreous gel is mainly composed of water and while there may be an association between hydration status and contraction of the vitreous gel leading to traction on the retina (resulting in flashes of light), this has not been studied well. Currently, there is no research examining the effects of hydration status directly on the vitreous gel. Gradual hydration is best for your eyes and general health. Some individuals are at risk of high eye pressure after drinking larger volumes of water over a short period of time (this can happen with only a liter of water if consumed rapidly).

1. **Will a corneal transplant worsen my eye floaters?**

- **Answer:**
- Eye floaters are caused by shadows cast by the debris inside the back part of the eye. A corneal transplant does not affect that area directly. But a transplant can hasten the aging process inside the eye that leads to a posterior vitreous detachment (PVD). A PVD—which is a normal part of aging and happens to most people have by age 70—may result in increased floaters. But if a transplant is needed, the visual benefit of the surgery would typically outweigh a potential increase in floaters. If floaters are excessively bothersome, there are surgical procedures that can reduce floaters.

1. **Is there any connection between floaters/flashes and vertigo?**

- **Answer:**
- The vertigo is likely not linked to the flashes and floaters in your eye unless they disrupt your vision enough to become unbalanced and unsteady on your feet. You should talk to your doctor about the vertigo and have a dilated eye examination with your ophthalmologist to make sure there is no problem in the back of your eye like a torn retina.

1. **I have many floaters and am considering laser treatment for their removal. What are the pros and cons for this surgery?**

- **Answers :** There are some who are treating with laser bursts. These bursts tend to break up the floaters. There are few "pros" to such surgery. If you are lucky, the floaters may become small enough to drift out of the line of sight.
- The "cons" are many. The floaters may be made worse because there are more of them, or your surgeon may not be able to find the ones that bother you most. More importantly, you will have the risk of retinal tears, retinal swelling or edema, and even retinal detachment. Glaucoma, or too much pressure in the eye, is also possible. These conditions can be blinding, although generally these conditions are treatable. There are very few retinal surgeons who perform this procedure for the reasons mentioned above.

1. **Are floaters normal in children?**

**•Answer:**

**•**Spots in a young child's vision are occasionally reported. They can range from seeing dust in a beam of light to more serious ocular conditions. You should ask about any problem with vision for usual activities. In general, these symptoms should be evaluated by an ophthalmologist to exclude any serious eye conditions.

1. **In the case of a detached retina or virtual separation, are the light flashes also seen when the eyes are closed?**

- When there is a posterior vitreous detachment or retinal detachment most people notice the flashes whether the eyes are opened or closed.

1. **After your vitreous (jelly-like material inside the eye) has detached completely, can new floaters still arise from the now-detached vitreous?**

- Yes, new floaters are possible after the vitreous has detached. In fact, the floaters typically change over time. Some larger floaters may break up and other new floaters may become apparent. This is part of the continuing changes in the vitreous gel after detachment from the retina.

1. **I have what appear to be floaters in my eye, but the doctor cannot see them. Is it possible that I am very sensitive to them, but they cannot be seen by the doctor?**

- Floaters can occur for different reasons. The most common cause for floaters is vitreous degeneration, which is a slow process of change in the vitreous gel in the back of the eye. As the gel liquefies and condenses over time, we may start to see dark spots or floaters in our vision. They can be subtle on examination and at times very noticeable to you, the patient, especially in brighter lighting when your pupil becomes very small. This happens because you are actually seeing the shadow of the opacity (floater) rather than the floater itself, which may be very small and not obvious on exam.

1. **I have had a small ring similar to a smoke ring that floats in various locations in my right eye. I thought floaters were black spots floating in the vision. Is this also a floater?**

- Yes, and it probably is the shadow of a "Weiss ring," which is a tiny piece of tissue on the back surface of the vitreous gel, which has separated from your optic nerve. You have probably had a "vitreous detachment". If your symptoms are quite new, you should have a thorough evaluation of your retina by an ophthalmologist quite soon.

**LASIK and Laser surgery / 10**

1. **Can I have LASIK if I am a glaucoma suspect?**

- **Answer:**
- Given otherwise healthy eyes this should not be a problem. Modern-day LASIK does not raise eye pressure much and it only does so for 15 to 20 seconds at most.

1. **How many times can a person have LASIK eye surgery safely?**

- **Answer:**
- The limit for laser surgery has more to do with the thickness of your cornea. Every time surgery is performed, the cornea becomes thinner. The safe amount that can be removed differs with every eye. Your surgeon should be able to determine this for you. Sometimes if the eye is too thin, it still may be possible to perform PRK (surface ablation).

1. **Can PRK surgery cause recurrent corneal erosion?**

- **Answer :** Five percent of patients develop recurrent corneal erosion syndrome (when the cornea’s outer layer loosens from the layer underneath, causing pain and blurred vision) after photorefractive keratectomy (or PRK, a type of refractive surgery). This can happen in the periphery where the corneal epithelium has been removed, but outside of the treatment zone. Because the epithelium (outermost layer of the cornea) is removed during PRK, microcysts can form between the corneal epithelium and the corneal stroma as it heals. In this case, the epithelium is not as tightly adherent as it should be, and patients can suffer from recurrent corneal erosions. Talk to your eye doctor about potential treatment remedies.

1. **Should I have LASIK if I have cataracts?**

- **Answer:**
- LASIK surgery is not advised if cataracts are affecting your vision. Moreover, having any amount of cataract means that the good vision gained from LASIK may be short lived as the cataract will continue to grow and eventually change your vision. It is actually common for LASIK to be done after cataract surgery to fine-tune the patient’s vision.

1. **Is it safe to get LASIK with pigment dispersion syndrome?**

- **Answer:**
- Neither pigment dispersion syndrome or ocular hypertension should have any effect on having successful LASIK surgery. But both should be evaluated by your eye surgeon before you have LASIK.

1. **If I had strabismus surgery as a child can I still have LASIK?**

- **Answer:**
- As long as your eyes are found to be healthy enough for LASIK, past strabismus surgery will not prevent you from having LASIK.

1. **Can LASIK surgery cause choroidal neovascular membrane?**

- **Answer:**
- No, LASIK should not have any effect on the retina. Before having LASIK, be sure to discuss any existing eye conditions with your surgeon so you can find out if LASIK is right for you.

1. **When is it OK to get pregnant after LASIK?**

- **Answer:**
- You can get pregnant at any time. The concern is that some patients develop dry eye during their pregnancy but if you have healed well at one month, you should be fine going forward.

1. **Is LASIK Possible With Keratoconus?**

- **Answer:**
- Keratoconus—even mild—is a contraindication to LASIK or PRK. However to help ensure that it does not progress in the future, I would suggest that you get a consult with a corneal specialist who is doing cross linking treatment that can inhibit progression in the future. Once this is done, it is possible to consider refractive surgery.

1. **I have a higher than normal optic disc C/D ratio. Would LASIK complicate monitoring and detection of glaucoma?**

- **Answer:**
- If you do not have glaucoma at this time, there really should be no increased risk to the surgery itself. There are established methods for calculating eye pressure after LASIK surgery. However, glaucoma today is mostly diagnosed with visual field testing and imaging of the nerve fiber layer of the back of the eye which are unchanged by these refractive surgery procedures.

**Amblyopia, Strabismus, and Lazy eye / 10**

1. **Can surgery help fix exophoria?**

- **Answer:**
- Typically, surgery is not needed for exophoria (when one eye drifts outward). Some patients with convergence insufficiency may be exophoric (intermittent drifting) at times and exotropic (continuous drifting) at others. Surgery can help such patients if eye exercises and prism do not. While these treatments frequently improve the drifting, they may not cure the problem.

1. **Is surgery possible for adults with amblyopia ?**

- **Answer :** If these conditions are not fixed as a child (before the age of 9- to 10-years-old), the vision loss remains permanent. So no surgery can be done to make the eye see normal vision in adulthood if the amblyopia was not treated as a child. Having said that, adults with strabismus amblyopia can have eye muscle surgery to straighten the eyes but it only improves cosmetic appearance and won't improve vision in the amblyopic eye.

1. **Why do patches help vision in weak eye while eye exercises don't?**

- **Answer:**
- Generally, eye exercises do not improve blurry vision or treat eye disease. In some cases, eye exercises may be useful for a problem called convergence insufficiency (when the eyes have trouble working together for near focus). Also, changing focus during near work with the “20-20-20 rule” may help with symptoms of digital eye strain. Ask your ophthalmologist if eye exercises can help you.
- An eye patch can help a weak eye in some children with amblyopia. Patching the better seeing eye works by forcing the weaker eye to see. The dedicated time using the weaker eye results in better development of vision in that eye.
- Patching a child who has not been assessed by an ophthalmologist can lead to unnecessary treatment and could actually cause the eye being patched to become weak. So, patching should not be started by a parent without direction from your ophthalmologist.

1. **Will my IOL correct my crossed eye?**

- **Answer:**
- There is no way to tell without your ophthalmologist measuring this prior to doing any surgery. You may need to see a strabismus (eye muscle) specialist to evaluate this.

1. **Did my contact lenses stop correcting my crossed eyes?**

- **Answer:**
- Accommodative esotropia (when eyes cross inward while focusing to see up close) is a condition that usually happens in early childhood but can also re-emerge later in life. The most important step is to have a complete exam by an ophthalmologist with "cycloplegic refraction." Your glasses or contact lenses are possibly undercorrecting your farsighted vision. When this happens, it can result in crossing.
- With cycloplegic refraction, eye drops are used which keep your eyes dilated most of the day. This type of exam can reveal latent or hidden farsightedness.

1. **Can my nerve damage be related to my strabismus?**

- **Answer:**
- The nerve damage that you describe related to the lip corner and eyelid closure is unlikely to be directly related to the eye misalignment. However, the same original issue that caused the nerve damage could have caused damage to different nerves that control the eye alignment.

1. **If I had strabismus surgery as a child, can I still have LASIK?**

- **Answer:**
- As long as your eyes are found to be healthy enough for LASIK, past strabismus surgery will not prevent you from having LASIK.

1. **Will patching my eye with AMD harm my eyes?**

- **Answer:**
- Normally we recommend avoiding prolonged covering of one eye since it can lead to strabismus or crossed eyes.
- For short periods of time and during specific tasks you could consider covering one eye, but again, we generally recommend against prolonged patching or covering of one eye. However, covering the eye does not damage one eye or the other eye or affect the macular degeneration. Talk with your ophthalmologist about your visual distractions and their recommendations for reducing them.

1. **Could high altitude affect esotropia?**

- **Original question :** My 19-year-old daughter's right eye is wandering towards her nose. It has gotten worse since she's been in college in Colorado. Could the high altitude have anything to do with this? She is scheduled for an MRI next week to see why this eye has started to wander. What else could this be from?
- **Answer:**
- The altitude is not an issue with respect to the strabismus and I hope she is enjoying college. As for the cause of the inward wandering eye which we call esotropia, if there is a history of strabismus then there is no worry about something serious. Some patients with childhood-onset strabismus lose control of their alignment as they age. Others who are farsighted and stopped wearing glasses in high school might benefit by resuming glasses or contact lenses. If this condition is new, then an MRI is reasonable along with a complete medical evaluation to search for the cause.

1. **My husband obtained a lazy eye after an eye injury and multiple surgeries.** **Now our newborn was born with one and it’s the same eye. Is My Daughter's Lazy Eye Inherited?**

- **Answer:**
- While some cases of strabismus or eye misalignment appear to be inherited, there is no reason to believe that your husband’s injury and eye condition were inherited by your daughter.
